# Awareness of Sexual Partner’s HIV Status Among Men Who Have Sex With Men in China: Cross-Sectional Survey Study

**DOI:** 10.2196/66139

**Published:** 2025-01-13

**Authors:** Qian Ma, Tingting Jiang, Wanjun Chen, Shaoqiang Jiang, Jinlei Zheng, Hui Wang, Lin He

**Affiliations:** 1 Department of Rheumatology Immunology & Allergy, The Children’s Hospital, Zhejiang University School of Medicine, National Clinical Research Center for Child Health, Hangzhou, China; 2 Zhejiang Provincial Center for Disease Control and Prevention, Hangzhou, China; 3 Coastal Service Center, Qiantang District, Hangzhou, Zhejiang, China

**Keywords:** human immunodeficiency virus, HIV, serostatus disclosure, pre-exposure prophylaxis, PrEP, men who have sex with men, MSM, web-based survey, HIV awareness

## Abstract

**Background:**

Men who have sex with men (MSM) constitute a significant proportion of individuals living with human immunodeficiency virus. Over the past few years, China has implemented various strategies aimed at increasing the rate of HIV testing and reducing HIV transmission among MSM. Among these, the disclosure of HIV serostatus is an effective prevention strategy.

**Objective:**

This study aimed to assess HIV serostatus disclosure and identify factors associated with awareness of sexual partners’ HIV status among MSM to provide a scientific basis for promoting HIV testing and reducing HIV transmission.

**Methods:**

A cross-sectional study based on a large-scale web-based survey was conducted among MSM in Zhejiang province, China, between July and December 2023. MSM who were HIV-negative or had an unknown HIV status were recruited from the Sunshine Test, a web-based platform that uses location-based services to provide HIV prevention services. Participants were required to complete a questionnaire on demographic characteristics, sexual behavior, rush popper use, awareness of sexual partners’ HIV status, and knowledge of pre-exposure prophylaxis (PrEP) and postexposure prophylaxis (PEP). A multinomial regression model was used to identify the factors associated with awareness of sexual partners’ HIV status.

**Results:**

A total of 7629 MSM participated in the study, with 45.2% (n=3451) being aware, 35.4% (n=2701) being partially aware, and 19.4% (n=1477) being unaware of their sexual partner’s HIV status. The multinomial logistic regression analysis revealed the following results. Compared to those who were unaware of their sexual partner’s HIV status, participants who were students (adjusted odds ratio [aOR] 1.43, 95% CI 1.09‐1.86), had a monthly income of more than US $1400 (aOR 1.36, 95% CI 1.03‐1.80), had insertive anal sex (aOR 1.35, 95% CI 1.12‐1.63), had only male sexual partners (aOR 1.53, 95% CI 1.28‐1.82), had 1 sexual partner in the past 3 months (aOR 2.36, 95% CI 2.01‐2.77), had used condoms for the past 3 months (aOR 1.72, 95% CI 1.33‐2.22), had frequently used rush poppers in the past 3 months (aOR 2.27, 95% CI 1.81‐2.86), were aware of HIV PrEP (aOR 2.04, 95% CI 1.68‐2.48), were aware of HIV PEP (aOR 1.69, 95% CI 1.39‐2.06), used mail reagent self-testing (aOR 1.19, 95% CI 1.04‐1.36), and had previously undergone HIV testing (aOR 1.40, 95% CI 1.16‐1.69) were associated with increased odds of being aware of their sexual partner’s HIV status.

**Conclusions:**

Overall, 45.2% of MSM who were HIV-negative or had an unknown status were aware of their sexual partners’ HIV status in China. We suggest strengthening targeted interventions through web-based platforms and gay apps to promote the disclosure of HIV serostatus and reduce HIV transmission among MSM.

## Introduction

Men who have sex with men (MSM) constitute a significant proportion of individuals living with human immunodeficiency virus. The overall HIV prevalence among MSM in China has remained at 8% for the past several years [1]. The annual number of newly diagnosed HIV infections is >100,000, with 25.7% of new cases resulting from homosexual transmission in 2023 [2]. The Joint United Nations Program on HIV/AIDS (UNAIDS) proposed a 95-95-95 target by 2030 [3]. The target was to achieve 95% of patients infected with HIV diagnosed, 95% of diagnosed patients treated, and 95% of treated patients achieving viral suppression. In China, the second and third 95% targets (95.1% and 97.3% in 2023, respectively) were achieved with the implementation of a free treatment policy and lifelong follow-up, but only 84.3% of patients have been diagnosed [2]. There are challenges to achieving the first 95% target in China. Scaling up HIV screening for MSM is an important means to achieving the first 95% target, but there are still major challenges. Data from 2023 showed that only 88% of MSM had ever been tested for HIV [4]. Various testing strategies, including regular testing, self-testing, web-based mail testing, and serostatus disclosure, have been promoted to expand HIV testing among MSM [5].

The serostatus disclosure strategy is an effective measure for reducing and controlling HIV transmission. Obermeyer et al [6] have defined HIV serostatus disclosure as “the process of revealing a person’s HIV status, whether positive or negative.” Serostatus disclosure requires a person to ask or know their partner’s HIV status before having sex and to only have sex if they have the same HIV status or use protective measures. Self-disclosure is the most common form of disclosure, which requires a person to share information about his or her HIV status directly with another person. In recent years, serostatus disclosure strategies have been the focus of intervention efforts among people with a high risk of HIV infection and have played an important role in HIV transmission risk reduction. One study showed that disclosure was associated with a 45% reduction in HIV transmission [7]. Empirical studies have shown that disclosure of HIV serostatus by people living with HIV can reduce the risk of HIV transmission by 17.9%‐40.6% compared to no disclosure [8]. Disclosure to sexual partners could also increase the HIV testing rate and reduce transmission risk behaviors [9]. A benefit of disclosing HIV status is that it empowers sexual partners to make shared decisions about how to protect their health while engaging in an active sexual life [10]. Prevalence of HIV disclosure among MSM living with HIV ranges from 12% to 53% [11]. Accordingly, most HIV infections occur when there is a lack of knowledge of the HIV-positive status of the other partner, coupled with a low uptake of preventive measures and inconsistent condom use [12]. Common barriers to disclosure among MSM were a lower perceived risk of HIV infection, history of sexually transmitted infections, engagement in receptive sex, and having sex with casual partners [13-15]. Patients with HIV viral suppression have a lower disclosure rate [16], as it is now established that there is no risk of HIV transmission when the viral load is undetectable [17,18]. Other studies have shown that being unmarried, living in rural formal areas [15], being aged 50 years and over [19], lacking family support [20], poverty, having multiple sexual partners, and stigma [21,22] lead to a lower rate of HIV serostatus disclosure. In addition, substance use prior to sexual encounters is less likely in lower-risk locations compared to highest-risk locations (ie, bathhouse and public sex environment) [23].

In China, individuals infected with HIV are required by law to disclose an HIV infection to their spouses. Furthermore, more than 95% of patients’ spouses know their HIV status, and more than 92% of spouses have been tested for HIV yearly [24]. Therefore, China’s HIV serostatus disclosure strategy is predominantly promoted among high-risk populations susceptible to HIV infections, including MSM. This is achieved by implementing an HIV serostatus disclosure strategy with the aim of expanding HIV testing coverage, enabling the early detection of individuals infected with HIV, and subsequently reducing HIV transmission among MSM. However, there are barriers to implementing this strategy, including the prevalence of multiple sexual partners in the MSM population, fear of being rejected by sexual partners, and the personal privacy of one’s infection status. In addition, the implementation of the “undetectable equals untransmittable” strategy means that many people living with HIV either do not disclose or do not ask about the HIV status of their sexual partners. Therefore, this could be a factor in a partner’s decision to use a condom during sexual intercourse [10]. When implementing the serostatus disclosure strategy, MSM who are HIV-negative or unaware of their infection status are advised to ask their partners about their HIV infection status before sex, to self-test for HIV, and to take appropriate safety measures according to their HIV status. It has also been shown that HIV-negative MSM become more aware of their partners’ serostatuses over time [25].

With the development of the digital economy, an increasing number of MSM are looking for sexual partners through the internet and dating apps. This study shows that the level of HIV knowledge among MSM can be improved and HIV testing can be expanded by relying on the internet. Consequently, web-based interventions and HIV testing among MSM populations, including web-based counseling and mailing of HIV self-test kits, are becoming increasingly common. A previous meta-analysis showed that using mobile health technology could increase the linkage to care among MSM using HIV self-testing [26].

Zhejiang province is economically developed, with an active digital economy. Approximately 66,000 HIV tests were performed among MSM in 2023, accounting for 10% of the national proportion [2], and over 70% of HIV testing among MSM in Zhejiang province relied on the internet. The promotion of pre-exposure prophylaxis (PrEP) and postexposure prophylaxis (PEP) prevention services in China began in 2020. To work toward achieving the target of 95% of HIV infections diagnosed globally and in China, a large-scale, web-based cross-sectional study was conducted. This study aimed to assess the awareness of a sexual partner’s HIV status before engaging in sexual intercourse among MSM, especially for those who were not infected with HIV or did not know their infection status, which could be causing an information gap among MSM in China [13]. In the context of PrEP and PEP prevention services and the implementation of the “undetectable equals untransmittable” strategy, this study aimed to assess HIV serostatus disclosure and identify factors associated with awareness of sexual partners’ HIV status among MSM to provide a scientific basis for promoting HIV testing and reducing HIV transmission.

## Methods

### Sampling

A cross-sectional survey was conducted between July and December 2023. The survey was based on a large web-based survey of MSM. To determine the required sample size, we used the following formula:


$$n=Z_{\alpha /2}\;P\left(1-P\right)$$

The *α* level was .05, *P* was estimated as 20%‐30%, and δ was 0.05 resulting in a minimum sample size of 1329.

### Study Participants

The following inclusion criteria were used to recruit MSM participants: (1) at least 16 years of age, (2) having had sex with men in the past year, (3) HIV-negative or unknown HIV status, and (4) living in Zhejiang province. Participants were excluded if they had previously received 2 or more HIV tests.

### Participant Recruitment and Data Collection

Sunshine Coast Public Welfare is a social service organization that uses the internet to provide HIV outreach and testing services to the MSM population. Through the use of location-based services, Sunshine Coastal Public Welfare established a digital HIV prevention service. MSM in Zhejiang province could apply for free HIV-testing services by visiting the official account of the Sunshine Test on WeChat (a popular communication software in China). There were two ways for MSM to access HIV testing services: they could choose to have the test reagent sent by courier, or they could choose to self-test after receiving the reagent. Alternatively, they could choose offline services, where volunteers in their neighborhood provided HIV testing services, or they could visit a nearby voluntary counseling and testing site. MSM who applied for HIV testing services through the Sunshine Test were required to complete a questionnaire that covered demographic characteristics such as age, marital status, and education level, as well as sexual behavior characteristics such as sexual roles, number of sexual partners, use of rush poppers, sexual history, PrEP use, PEP use, and HIV testing history. All the questionnaires were completed using the Sunshine Test [4].

### Statistical Analysis

For descriptive analyses, categorical variables were presented as frequencies and proportions, whereas continuous variables were presented as medians and IQRs or means and SDs. Differences in general demographic characteristics of the study population were compared using a *χ*^2^ test. The main outcome was awareness (participants knowing all of their sexual partners’ HIV statuses) and partial awareness (participants only knowing some of their partners’ HIV statuses) of their sexual partners’ HIV status, with those who were unaware of their sexual partner’s HIV status being used as the reference group. Multinomial logistic regression was used to identify the factors associated with the awareness and partial awareness of a partners HIV status compared to the unaware group. Factors from the univariate analysis were included in the multinomial regression models. The α level was .05 and *β* was .1. All statistical analyses were performed using SPSS version 19.0 (IBM Corp).

### Ethical Considerations

This study was approved by the Zhejiang Provincial Center for Disease Control and Prevention (approval number 2022-011-01). Written informed consent was obtained from all participants prior to survey completion. Participants received free HIV testing and health counseling, and all study procedures were conducted in accordance with approved guidelines and regulations. Participants could dop out during any stage of the survey. All the data was anonymized no amount of compensation was provided.

## Results

**Figure 1.**
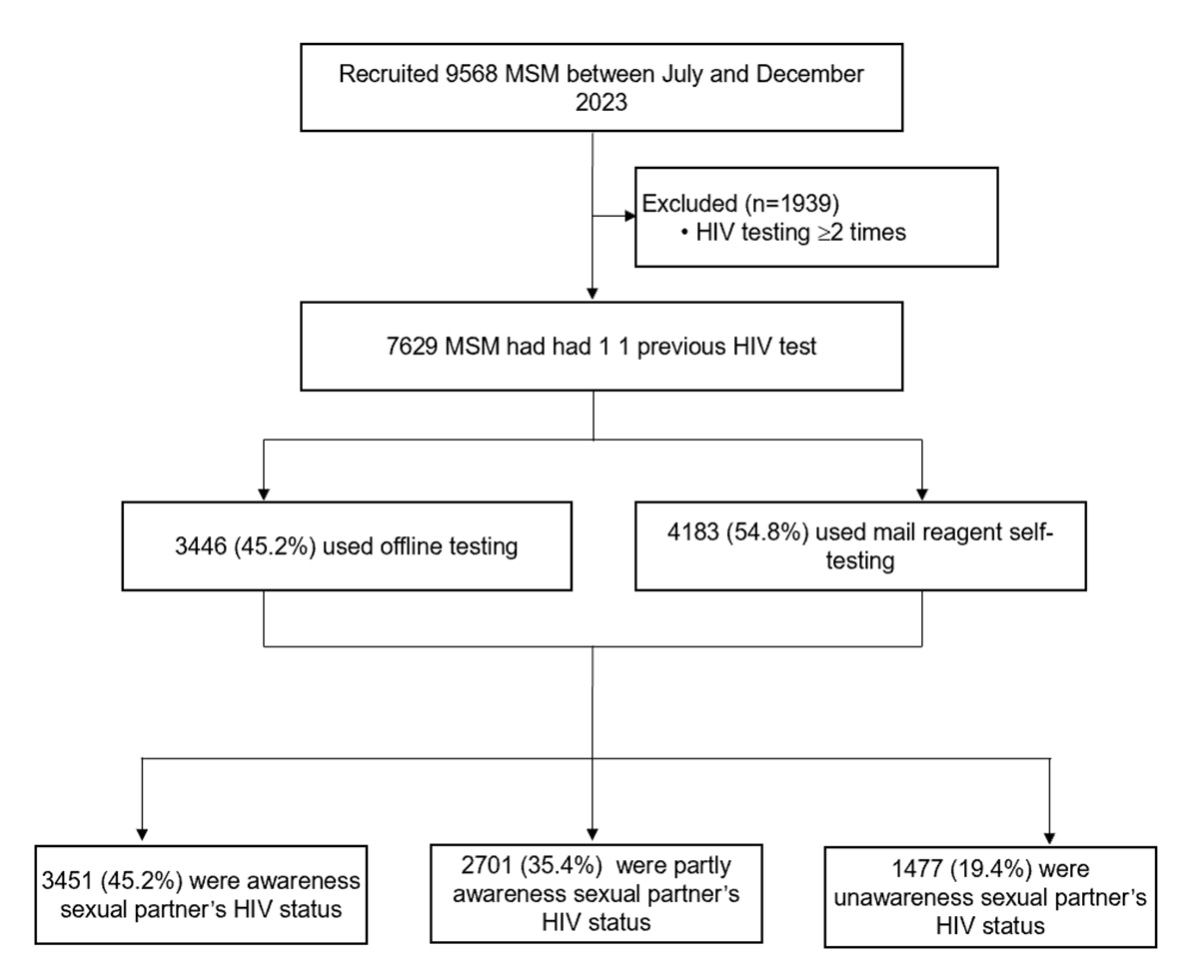
Study design.

A total of 7629 MSM participated in the study (Figure 1), and 64.9% (4956/7629) were aged less than 30 years. Among them, 50.7% (3869/7629) had a bachelor’s degree or higher, 16.4% (1254/7629) were students, and 39.1% (2985/7629) had a monthly income of less than US $700. The majority (78.2%, 5964/7629) had only male sexual partners, while 33.3% (2543/7629) had more than 2 sexual partners in the past 3 months. Additionally, 31.2% (2380/7629) reported using rush poppers during sexual activity. The awareness rates of PrEP and PEP were 76.2% (5816/7629) and 78.1% (5960/7629), respectively. Furthermore, 54.8% (4183/7629) had HIV testing using a mail reagent self-test, and 12.9% (982/7629) were undergoing HIV testing for the first time (Table 1).

**Table 1.** Social demographic and behavioral characteristics among men who have sex with men.

Variables	Total (n=7629), n (%)	Awareness of sexual partner’s HIV status			Chi-square (*df*)	*P* value
		Yes (n=3451)	Partially (n=2701)	No (n=1477)		
Age^a^ (years), n (%)					33.458 (4)	<.001
<20	430 (5.6)	185 (43)	136 (31.6)	109 (25.3)		
20‐29	4526 (59.3)	2143 (47.3)	1528 (33.8)	855 (18.9)		
≥30	2673 (35)	1123 (42)	1037 (38.8)	513 (19.2)		
Marital status, n (%)					35.719 (4)	<.001
Single	5997 (78.6)	2804 (46.8)	2032 (33.9)	1161 (19.4)		
Married	1388 (18.2)	558 (40.2)	571 (41.1)	259 (18.7)		
Divorced/separated	244 (3.2)	89 (36.5)	98 (40.2)	57 (23.4)		
Education, n (%)					77.958 (6)	<.001
High school and below	1851 (24.3)	683 (36.9)	736 (39.8)	432 (23.3)		
College	1909 (25)	898 (47)	639 (33.5)	372 (19.5)		
Bachelor’s degree	3145 (41.2)	1511 (48)	1099 (34.9)	535 (17)		
Master’s degree or above	724 (9.5)	359 (49.6)	227 (31.4)	138 (19.1)		
Occupation, n (%)					54.237 (6)	<.001
Student	1254 (16.4)	608 (48.5)	375 (29.9)	271 (21.6)		
Company employee	2843 (37.3)	1370 (48.2)	1009 (35.5)	464 (16.3)		
Freelance	1022 (13.4)	426 (41.7)	383 (37.5)	213 (20.8)		
Others	2510 (32.9)	1047 (41.7)	934 (37.2)	529 (21.1)		
Monthly income (US$), n (%)					38.718 (6)	<.001
<$350	1320 (17.3)	577 (43.7)	419 (31.7)	324 (24.5)		
$350-$699	1665 (21.8)	721 (43.3)	633 (38)	311 (18.7)		
$700-$1399	3259 (42.7)	1477 (45.3)	1178 (36.1)	604 (18.5)		
≥$1400	1385 (18.2)	676 (48.8)	471 (34)	238 (17.2)		
Sex roles, n (%)					131.721 (4)	<.001
Receptive anal sex	1750 (22.9)	796 (45.5)	675 (38.6)	279 (15.9)		
Insertive anal sex	2652 (34.8)	1319 (49.7)	949 (35.8)	384 (14.5)		
Both	3227 (42.3)	1336 (41.4)	1077 (33.4)	814 (25.2)		
Sex of partner, n (%)					284.131 (2)	<.001
Male only	5964 (78.2)	2852 (47.8)	2197 (36.8)	915 (15.3)		
Male and female	1665 (21.8)	599 (36)	504 (30.3)	562 (33.8)		
Number of sexual partners in the past 3 months, n (%)					748.589 (4)	<.001
0	1277 (16.7)	475 (37.2)	296 (23.2)	506 (39.6)		
1	3809 (49.9)	2124 (55.8)	1145 (30.1)	540 (14.2)		
≥2	2543 (33.3)	852 (33.5)	1260 (49.5)	431 (16.9)		
Condom use in the past 3 months, n (%)					681.686 (6)	<.001
Every time	4266 (55.9)	2171 (50.9)	1445 (33.9)	650 (15.2)		
Sometimes	2005 (26.3)	751 (37.5)	958 (47.8)	296 (14.8)		
No sex	874 (11.5)	288 (33)	168 (19.2)	418 (47.8)		
Never	484 (6.3)	241 (49.8)	130 (26.9)	113 (23.3)		
Ever used rush poppers during sexual behavior, n (%)					169.468 (4)	<.001
Never	5249 (68.8)	2355 (44.9)	1704 (32.5)	1190 (22.7)		
Occasionally	1312 (17.2)	536 (40.9)	601 (45.8)	175 (13.3)		
Often	1068 (14)	560 (52.4)	396 (37.1)	112 (10.5)		
History of STIs^b^, n (%)					7.553 (2)	.02
No	7428 (97.4)	3376 (45.4)	2612 (35.2)	1440 (19.4)		
Yes	201 (2.6)	75 (37.3)	89 (44.3)	37 (18.4)		
Awareness of HIV PrEP^c^, n (%)					336.193 (2)	<.001
No	1813 (23.8)	551 (30.4)	669 (36.9)	593 (32.7)		
Yes	5816 (76.2)	2900 (49.9)	2032 (34.9)	884 (15.2)		
Knowledge of HIV PEP^d^, n (%)					313.701 (2)	<.001
No	1669 (21.9)	528 (31.6)	579 (34.7)	562 (33.7)		
Yes	5960 (78.1)	2923 (49)	2122 (35.6)	915 (15.4)		
HIV testing pathway, n (%)					26.881 (2)	<.001
Offline testing	3446 (45.2)	1526 (44.3)	1165 (33.8)	755 (21.9)		
Mail reagent self-testing	4183 (54.8)	1925 (46)	1536 (36.7)	722 (17.3)		
HIV status, n (%)					7.114 (2)	.03
Positive	82 (1.1)	27 (32.9)	31 (37.8)	24 (29.3)		
Negative	7547 (98.9)	3424 (45.4)	2670 (35.4)	1453 (19.3)		
First time undergoing HIV testing, n (%)					59.378 (2)	<.001
No	6647 (87.1)	3024 (45.5)	2421 (36.4)	1202 (18.1)		
Yes	982 (12.9)	427 (43.5)	280 (28.5)	275 (28)		

a The median age was 27 (IQR 23-33).

b STI: sexually transmitted infection.

c PrEP: pre-exposure prophylaxis.

d PEP: postexposure prophylaxis.

Of the 7629 participants, 45.2% (3451/7629) were aware, 35.4% (2701/7629) were partially aware, and 19.4% (1477/7629) were unaware of their sexual partner’s HIV status. Factors associated with high awareness of their sexual partner’s HIV status included being 20‐29 years old (47.3%, 2143/4526), being single (46.8%, 2804/5997), having a high education level (49.6%, 359/724), having a high monthly income (48.8%, 676/1385), performing insertive anal sex (49.7%, 1319/2652), having one sexual partner in the past 3 months (55.8%, 2124/3809), using condoms in the past 3 months (50.9%, 2171/4266), frequently using rush poppers (52.4%, 560/1068), being aware of HIV PrEP (49.9%, 2900/5816) and PEP (49.0%, 2923/5960), and being HIV-negative (45.4%, 3424/7547). The *χ*^2^ test showed that all factors were associated with awareness of sexual partners’ HIV status (Table 1).

As presented in Table 2, multinomial logistic regression analysis showed that, compared to those who were unaware of their sexual partner’s HIV status, participants who were students (adjusted odds ratio [aOR] 1.43, 95% CI 1.09‐1.86), had a monthly income of more than US $1400 (aOR 1.36, 95% CI 1.03‐1.80), had insertive anal sex (aOR 1.35, 95% CI 1.12‐1.63), had only male sexual partners (aOR 1.53, 95% CI 1.28‐1.82), had 1 sexual partner in the past 3 months (aOR 2.36, 95% CI 2.01‐2.77), used condoms in the past 3 months (aOR 1.72, 95% CI 1.33‐2.22), frequently used rush poppers in the past 3 months (aOR 2.27, 95% CI 1.81‐2.86), were aware of HIV PrEP (aOR 2.04, 95% CI 1.68‐2.48), were aware of HIV PEP (aOR 1.69, 95% CI 1.39‐2.06), used mail reagent self-testing (aOR 1.19, 95% CI 1.04‐1.36), and were undergoing HIV testing for the first time (aOR 1.40, 95% CI 1.16‐1.69) were associated with increased odds of awareness of their sexual partner’s HIV status.

**Table 2.** Factors associated with awareness of sexual partner’s HIV status among men who have sex with men.

Variables	Partial awareness		Awareness	
	aOR^a^ (95% CI)	*P* value	aOR (95% CI)	*P* value
Occupation				
Student	1.05 (0.80‐1.39)	.71	1.43 (1.09‐1.86)	.009
Company employee	1.14 (0.96‐1.36)	.14	1.17 (0.99‐1.38)	.07
Freelance	1.01 (0.82‐1.25)	.93	1.01 (0.82‐1.25)	.91
Others	1.00 (reference)	—^b^	1.00 (reference)	—
Monthly income (US$)				
<$350	1.00 (reference)	—	1.00 (reference)	—
$350-$699	1.30 (1.01‐1.67)	.045	1.36 (1.03‐1.80)	.03
$700-$1399	1.11 (0.86‐1.41)	.43	1.36 (1.07‐1.73)	.01
≥$1400	0.97 (0.73‐1.30)	.85	1.36 (1.03‐1.80)	.03
Sex roles				
Receptive anal sex	1.00 (reference)	—	1.00 (reference)	—
Insertive anal sex	1.08 (0.89‐1.31)	.35	1.35 (1.12‐1.63)	.002
Both	0.92 (0.76‐1.10)	.45	1.02 (0.85‐1.23)	.82
Sex of partner				
Male only	1.39 (1.16‐1.66)	<.001	1.53 (1.28‐1.82)	<.001
Male and female	1.00 (reference)	—	1.00 (reference)	—
Number of sexual partners in the past 3 months				
0	0.84 (0.61‐1.14)	.27	1.73 (1.27‐2.32)	<.001
1	0.91 (0.78‐1.07)	.25	2.36 (2.01‐2.77)	<.001
≥2	1.00 (reference)	—	1.00 (reference)	—
Condom use in the past 3 moths				
Every time	1.91 (1.45‐2.52)	<.001	1.72 (1.33‐2.22)	<.001
Sometimes	2.57 (1.92‐3.45)	<.001	1.42 (1.08‐1.88)	.01
No sex	0.64 (0.42‐0.97)	.04	0.65 (0.44‐0.95)	.03
Never	1.00 (reference)	—	1.00 (reference)	—
Ever used rush poppers during sex behavior				
Never	1.00 (reference)	—	1.00 (reference)	—
Occasionally	1.63 (1.34‐1.98)	<.001	1.35 (1.10‐1.64)	.003
Often	1.77 (1.40‐2.24)	<.001	2.27 (1.81‐2.86)	<.001
Awareness of HIV PrEP^c^				
No	1.00 (reference)	—	1.00 (reference)	—
Yes	1.22 (1.00‐1.49)	.048	2.04 (1.68‐2.48)	<.001
Awareness of HIV PEP^d^				
No	1.00 (reference)	—	1.00 (reference)	—
Yes	1.56 (1.28‐1.91)	<.001	1.69 (1.39‐2.06)	<.001
HIV testing pathway				
Offline testing	1.00 (reference)	—	1.00 (reference)	—
Mail reagent self-testing	1.20 (1.04‐1.38)	.01	1.19 (1.04‐1.36)	.01
First time undergoing HIV testing				
No	1.59 (1.30‐1.93)	<.001	1.40 (1.16‐1.69)	<.001
Yes	1.00 (reference)	—	1.00 (reference)	—

a aOR: adjusted odds ratio.

b Statistical analysis was not done for the reference group.

c PrEP: pre-exposure prophylaxis.

d PEP: postexposure prophylaxis.

Multinomial logistic regression analysis showed that, compared to those who were unaware of their sexual partner’s HIV status, participants who had a monthly income of US $350-$699 (aOR 1.30, 95% CI 1.01‐1.67), only had male sexual partners (aOR 1.39, 95% CI 1.16‐1.66), used condoms in the past 3 months (aOR 1.91, 95% CI 1.45‐2.52), frequently used rush poppers in the past 3 months (aOR 1.77, 95% CI 1.40‐2.24), were aware of HIV PrEP (aOR 1.22, 95% CI 1.00‐1.49), were aware of HIV PEP (aOR 1.56, 95% CI 1.28‐1.91), used mail reagent self-testing (aOR 1.20, 95% CI 1.04‐1.38), and were undergoing HIV testing for the first time (aOR 1.59, 95% CI 1.30‐1.93) were associated with increased odds of partial awareness of their sexual partner’s HIV status (Table 2).

## Discussion

### Principal Findings

This study described the awareness of sexual partners’ HIV status and its associated factors among MSM with an HIV-negative or unknown status in Zhejiang province, China. Based on data from 7629 participants, we found 45.2% (n=3451) of participants were aware of their sexual partner’s HIV status and 19.4% (1477) were unaware of their partner’s HIV status. Our results show higher percentages compared with studies from Guangzhou, China, which revealed that 36% of MSM were aware of the HIV serostatus of all their sexual partners [27], 37.8% of MSM had received their partner’s HIV testing report before engaging in sex [28], and 30.4% of MSM asked all partners about their HIV status [13]. A previous study from the United States found that 55% of participants consistently disclosed their HIV status to their sexual partners, 30% inconsistently disclosed, and 15% did not disclose [29], and these rates were lower than the disclosure rates of 61.2% in Korea [30] and 64.3% in Thailand [31]. The HIV serostatus disclosure strategy has proven effective in preventing and reducing HIV transmission. HIV prevalence among the MSM population has remained at 8% and only 84.3% of HIV-positive people have been diagnosed. We recommend the continued promotion and implementation of serostatus disclosure strategies among MSM, especially proactively asking about their sexual partners’ HIV status, taking appropriate protective measures based on HIV status, reducing HIV transmission, and undergoing early diagnosis testing for HIV.

Consistent with previous studies [28,32], we found that MSM who had undergone HIV testing were more likely to obtain their sexual partners’ HIV status. MSM who engage in HIV testing and seek their partners’ HIV status may reflect a heightened awareness and proactive approach toward HIV management. Individuals who have already undergone HIV testing may be better informed about the importance of knowing their partners’ HIV status, thereby reducing HIV transmission risks and promoting safer sexual practices. We found that MSM who used mail reagent self-testing had higher awareness of their partner’s HIV status. This suggests that HIV testing through web-based platforms or gay apps could increase HIV serostatus disclosure among MSM [33]. These findings suggest that interventions aimed at increasing HIV testing rates among MSM could indirectly enhance partner disclosure and engagement in HIV-prevention strategies. It supports the need for continued efforts to promote regular HIV testing and web-based self-testing to increase the HIV serostatus disclosure rate. Our study provides evidence that understanding and engaging in HIV testing behaviors may contribute to HIV serostatus disclosure among MSM.

MSM who were aware of HIV PrEP and PEP were more likely to obtain their sexual partners’ HIV status. A previous study showed that awareness of and access to PrEP might reduce the need and motivation to discuss HIV status with partners because they may assume that it is the partner’s responsibility to use PrEP to protect themselves from HIV infection [13]. PrEP users expressed significantly greater openness to serodifferent partnering than participants who had never used PrEP [34], and MSM were more likely to have sex with a person who disclosed being on PrEP [35]. Of those who had not disclosed their status to their partners, 71% stated PrEP would encourage them to have sex [36]. However, another study showed that PrEP awareness was not associated with HIV serostatus disclosure among Chinese MSM [13]. Knowledge of PrEP and PEP may drive more thorough discussions on HIV status, aligning with a proactive approach to reducing transmission risks. Against the background of a 78% awareness of PrEP and PEP in China, we recommend targeted interventions to increase awareness of PrEP and PEP, thereby increasing the rate of partners’ HIV testing and disclosure.

Consistent with a previous study [37], we found that MSM who used condoms and had fewer sexual partners were more likely to obtain the HIV status from their partners. MSM who adhere to consistent condom use and have fewer sexual partners often exhibit a heightened awareness of HIV risks and a commitment to preventing transmission. Findings showed that HIV status disclosure was significantly associated with lower rates of condomless sex compared to nondisclosure [33,38,39]. The probability of HIV exposure through condomless anal intercourse was substantially lower after serostatus disclosure than after nondisclosure [22]. One study showed that MSM engaging in receptive sex were less likely to ask their partners about their HIV status [13]. MSM with an HIV-negative or unknown status have a lower proportion of sexual events with a partner with an unknown HIV status [25]. Studies have demonstrated that MSM who disclose their HIV status are more likely to practice safer sexual behavior with HIV-infected partners than with partners with an HIV-negative or unknown status [23]. These findings highlight the importance of promoting consistent condom use and supporting MSM in maintaining low partner numbers to enhance the disclosure of HIV serostatus.

Moreover, we found that MSM who often used rush poppers had high awareness of their partners’ HIV status. Similar to other studies, drug use was independently associated with disclosure [40]. The possible reasons were as follows. First, MSM who used rush poppers had a higher rates of previous HIV testing, which was associated with greater awareness of their partner’s HIV status [4]. Second, due to the high use of rush poppers among MSM, rush popper use increases the risk of unprotected sexual behavior [39] and HIV transmission [41]. Targeted interventions were conducted for rush users among MSM [42], including reducing rush popper use, condom promotion, HIV serostatus disclosure, and other measures, such as knowing the HIV status of sexual partners before sex. We suggest that targeted interventions should be continued among MSM who use rush poppers, and this approach could help further improve the proportion of HIV serostatus disclosure.

### Limitations

This study has some limitations. First, the study used routine surveillance questionnaires, which may not have captured all factors associated with HIV serostatus disclosure in previous studies. For example, the questionnaire only investigated the awareness of PrEP but did not investigate whether they had used it, and previous studies showed that the use of PrEP could also improve the rates of HIV serostatus disclosure [35]. Second, the study focused on participants actively asking about their sexual partners’ HIV status and did not investigate whether the participants disclosed their HIV status to their sexual partners. Finally, the study recruited MSM from web-based applications, 64.9% of whom were under 30 years old. It did not include a few MSM who were over 50 years of age. Previous studies have shown that this population has lower HIV serostatus disclosure [19]. Therefore, the results of this study apply only to MSM who rely on the internet.

### Conclusion

The rate of awareness of sexual partners’ HIV status is low among MSM with an HIV-negative or unknown status in China. We suggest strengthening targeted interventions through web-based platforms and dating apps to promote HIV serostatus disclosure among MSM. Future studies could explore strategies to actively ask for a sexual partner’s HIV status among underrepresented demographics, such as older MSM and those who used PrEP, ultimately helping to improve the HIV disclosure rate and reduce HIV transmission.

## Abbreviations

MSM: men who have sex with men
STI: sexually transmitted infections
PEP: postexposure prophylaxis
PrEP: pre-exposure prophylaxis
aOR: adjusted odds ratio
UNAIDS: Joint United Nations Program on HIV/AIDS (UNAIDS)
