# Correction: Awareness of Sexual Partner’s HIV Status Among Men Who Have Sex With Men in China: Cross-Sectional Survey Study

**DOI:** 10.2196/101200

**Published:** 2026-07-31

**Authors:** Qian Ma, Tingting Jiang, Wanjun Chen, Shaoqiang Jiang, Jinlei Zheng, Hui Wang, Lin He

**Affiliations:** 1 Department of Rheumatology Immunology & Allergy, The Children’s Hospital, Zhejiang University School of Medicine, National Clinical Research Center for Child Health, Hangzhou, China; 2 Zhejiang Provincial Center for Disease Control and Prevention, 3399 Bin Sheng Road, Binjiang District, Hangzhou, China, 86 057187115106; 3 Coastal Service Center, Qiantang District, Hangzhou, Zhejiang, China

In “Awareness of Sexual Partner’s HIV Status Among Men Who Have Sex With Men in China: Cross-Sectional Survey Study” [1], the authors made one correction.

Affiliation 1 has been changed from the following:

Department of Rheumatology Immunology & Allergy, Zhejiang University School of Medicine, Hangzhou, China

The affiliation now reads:

Department of Rheumatology Immunology & Allergy, The Children’s Hospital, Zhejiang University School of Medicine, National Clinical Research Center for Child Health, Hangzhou, China

The correction will appear in the online version of the paper on the JMIR Publications website together with the publication of this correction notice. Because this was made after submission to PubMed, PubMed Central, and other full-text repositories, the corrected article has also been resubmitted to those repositories.
